# Edinburgh Postnatal Depression Scale Factor Structure and Invariance in Fathers Across the First Two Postnatal Years: Evidence for a Three-Factor Model and Elevated Screening Positivity in Year 2

**DOI:** 10.1177/15579883261454722

**Published:** 2026-06-10

**Authors:** Jingyi Wang, Michael B. Wells

**Affiliations:** 1 Division of Social Science, The Hong Kong University of Science and Technology, China; 2 Department of Women’s and Children’s Health, Karolinska Institutet, Stockholm, Sweden

**Keywords:** fathers, Edinburgh Postnatal Depression Scale, factor structure, confirmatory factor analysis, measurement invariance, Sweden

## Abstract

The dimensional structure of the Edinburgh Postnatal Depression Scale (EPDS) in fathers remains debated, and it is unclear whether scale properties are comparable as children age through the first 2 years. This study aims to examine the EPDS factor structure and measurement invariance across infant age bands among Swedish fathers. We analyzed 2,318 EPDS assessments from 1,615 fathers when their child was 0 to 24 months (0–6: *n* = 597; 7–12: *n* = 701; 13–18: *n* = 485; 19–24: *n* = 535). Competing confirmatory factor analyses compared one-, two-, and three-factor models within each age band. We then tested measurement invariance across bands. EPDS cut-off ≥10 described screening-positive proportions. A three-factor model: anhedonia (items 1–2), anxiety (3–6), and depressive affect (7–10) outperformed one- and two-factor alternatives across all age bands, with acceptable-to-good fit. Latent factors were positively and substantially inter-correlated. Strict measurement invariance was supported across age bands, indicating that mean-level comparisons are defensible as children age. Using EPDS ≥ 10, the proportion screening positive was 16.75% (0–6 months), 20.26% (7–12), 26.39% (13–18), and 22.99% (19–24). Internal consistency for the total scale was good across bands. In a national cohort of Swedish fathers across the first two postnatal years, the EPDS showed a replicable three-factor structure and strict invariance across child-age bands, supporting valid comparisons over time. Screening yields non-trivial prevalence beyond 12 months, underscoring the value of father-inclusive screening into the second postnatal year.

## Introduction

Depression is a leading cause of disability worldwide, and depression plus anxiety cost the global economy ~US$1 trillion annually in lost productivity, underscoring the public health and economic stakes of accurate identification (Cuijpers et al., 2023). Paternal depression across infancy is prevalent, with pooled estimates in the first postnatal year indicating rates of approximately 8.4% to 10.4% (Cameron et al., 2016; Paulson & Bazemore, 2010; Rao et al., 2020). Family impacts include elevated risks of child emotional and behavioral difficulties extending into childhood and adolescence (Sweeney & MacBeth, 2016), adolescent depression (Gutierrez-Galve et al., 2019), poorer child socio-emotional outcomes via family processes such as relationship strain (Gutierrez-Galve et al., 2015), and co-occurrence with maternal depression within couples (Paulson & Bazemore, 2010). These epidemiologic and family-level considerations underscore the need to develop and validate robust screening measures for paternal depression and to better characterize how depressive symptoms and their patterns present and vary across fathers and the postnatal period (Cockshaw et al., 2023; Paulson & Bazemore, 2010).

Prevalence of paternal postnatal depression varies as infants age, with meta-analyses indicating the highest rates around 3 to 6 months postpartum (Cameron et al., 2016; Paulson & Bazemore, 2010; Rao et al., 2020). Maternal depression tends to peak earlier in the first 3 months and maternal and paternal depression are moderately correlated, which supports a plausible temporal lag in fathers as roles and routines consolidate after birth (Paulson & Bazemore, 2010). Research finds that paternal depression symptoms occur across multiple time bands within the first year, arguing against restricting screening to the earliest postpartum weeks (Cameron et al., 2016; Rao et al., 2020). Critically, in mothers, a recent meta-analysis shows that depression in the second postpartum year (13–24 months) is similar to the first year, indicating that risk does not resolve at 12 months (Hellyer et al., 2025). For fathers, depression occurs through toddlerhood (Cockshaw et al., 2023) and into early childhood (Tichovolsky et al., 2018). Conceptually, the 12- to 24-month period often involves transitions to regular childcare and renewed or intensified employment in dual-earner families, conditions linked to greater work–family conflict and higher depressive symptom burden among fathers. In some systems, later scheduling of paternal caregiving leave can also coincide with these pressures, aligning with evidence that parental-leave environments relate to parental mental health (Heshmati et al., 2023). These considerations motivate assessment of paternal depression beyond the first year and set up the need to evaluate whether depression symptom scales can be interpreted consistently for fathers across child age bands.

The Edinburgh Postnatal Depression Scale (EPDS) is widely used for postnatal screening and has been validated for use with fathers (Shafian et al., 2022). Foundational validations in men indicate workable performance characteristics for detecting probable major depression (Edmondson et al., 2010; Matthey et al., 2001), and subsequent synthesis in fathers suggests that optimal paternal cut-offs are often lower than female thresholds (commonly 7–10 vs. ≥11–13), while maintaining acceptable sensitivity and specificity (Shafian et al., 2022). At the same time, authors caution about limitations relevant to men, including possible under-capture of male-typical depressive presentations and context-dependent predictive values in low-prevalence settings (Matthey & Agostini, 2017), as well as an ongoing debate about EPDS dimensionality in paternal samples. For the EPDS in mothers, repeated analyses support a three-factor structure with anhedonia, anxiety, and depressive affect, alongside evidence for a strong general factor that explains shared variance (Coates et al., 2017; Reichenheim et al., 2011). Evidence is less consistent in fathers, in part because published factor-analytic results depend on estimator choice and item handling. Given the methodological differences, direct comparisons across studies are difficult as fit indices and reliability estimates are best interpreted within study rather than across studies. For example, in father samples, early work reported two factors rather than three, with one factor capturing anhedonia and another capturing a broader cluster of depression symptoms that includes anxiety and unhappiness, and with Item 10 showing minimal performance that led to its exclusion in a population-based study at about 3 months postpartum (Massoudi et al., 2013). In contrast, an Italian study that enrolled antenatal (~30 weeks), immediate postnatal (1–4 weeks), and non-perinatal fathers retained Item 10 yet still found a two-factor solution by factor analyses and documented very low endorsement of Items 9 and 10 (Loscalzo et al., 2015). In a large cohort of fathers assessed at 18 weeks’ gestation, 8 weeks, 8 months, and 21 months postpartum, a three-factor EPDS model fit better than one- or two-factor alternatives and partial non-invariance was observed for Item 9 by gender (Cockshaw et al., 2023).

Despite these caveats, higher paternal EPDS scores predict clinically meaningful outcomes, including poorer father–infant bonding and lower coparenting quality in prospective analyses (Mumin et al., 2026; Wells & Jeon, 2023), and are linked with adverse child emotional and behavioral outcomes, underscoring relevance for family health (Sweeney & MacBeth, 2016). Consequently, clarifying the EPDS’s latent structure and measurement properties specifically in fathers across multiple postnatal age bands has clinical and research relevance. Establishing a well-fitting factor structure can also support domain-specific interpretation and referral (e.g., anhedonia, anxiety, and depressive-affect factors) when those dimensions are appropriate for fathers (Cockshaw et al., 2023). Testing measurement invariance helps ensure that score differences across time reflect true changes in depressive symptoms rather than measurement artifacts (Cheung & Rensvold, 2002; Putnick & Bornstein, 2016), and demonstrating equivalent measurement across age bands improves cross-study comparability and supports meta-analytic synthesis in paternal samples (Putnick & Bornstein, 2016; Shafian et al., 2022).

Thus, this study examines the EPDS’s factor structure and measurement invariance across child age bands in a national sample of Swedish fathers. The aim is to clarify which constructs the EPDS assesses in fathers and whether total and subscale scores can be meaningfully compared across groups and over time. Given that earlier studies in fathers have tended to support two-factor solutions (Loscalzo et al., 2015; Massoudi et al., 2013) whereas more recent work has supported three-factor models (Cockshaw et al., 2023), we compared the three-factor EPDS models against one-factor, two-factor (anhedonia + general distress; anxiety + depressive affect) EPDS models across four infant age bands (0–6, 7–12, 13–18, and 19–24 months). We then tested configural, metric, scalar, and residual invariance to determine whether observed score differences across child-age bands reflect true differences in depressive symptoms rather than measurement artifacts.

## Method

### Procedure

We used data from a longitudinal study of Swedish fathers that examined the professional and social support fathers received from pregnancy through early childhood. Fathers were recruited between December 2021 and February 2022 (T1) through targeted Facebook advertisements stratified by Swedish region to enhance national coverage. The advertisements featured either (a) an image of a father–infant dyad expressing satisfaction, accompanied by the text “Are you a new father and satisfied with your new life as a parent?” or (b) an image of a father–infant dyad expressing difficulty, accompanied by the text “Are you struggling with your parental role?” This strategy was used to oversample fathers with depression symptoms (Wells & Aronson, 2021; Yoon et al., 2019). The advertisements included a link to an online survey. In the T1 survey, fathers first completed questions about perceived professional and social support during the transition to parenthood. Fathers were then given the option to submit the survey or to complete additional measures, including an assessment of depression symptoms. Fathers could choose to remain anonymous or provide an email address for future follow-up surveys. Of the 2,420 fathers who participated in the survey, 1,589 completed the depression assessment at T1. Among the 2,420 fathers, 1,148 provided an email address and consented to future contact. Approximately 6 months later (T2; June 2022), fathers who had agreed to be recontacted were invited via email to complete a follow-up online survey. A total of 658 fathers participated at T2. About 1 year later (T3; July 2023–January 2024), fathers were again invited via email to complete another online survey. In total, 746 fathers participated in the T3 survey. The majority (over 96%) completed the T3 survey in July 2023, with a small proportion responding in subsequent months.

The current study was granted ethical approval by the Swedish ethical review board (dnr: 2018/889-31/5). An information letter was presented on the first page of the survey, outlining the voluntary nature of participation, the right to withdraw at any time, and secure data storage in accordance with the General Data Protection Regulation (GDPR). Fathers were informed that clicking “Next” indicated their informed consent to participate. No incentives were provided.

### Participants

Across the three waves, 1,654 fathers completed the depression measure at least once, yielding a total of 3,016 depression assessments. Given this study’s focus on fathers of children aged 0 to 24 months, only the 2,493 assessments conducted when the child was 24 months or younger were retained. These assessments were then categorized into four groups based on the child’s age: 0 to 6 months, 7 to 12 months, 13 to 18 months, and 19 to 24 months. Within each age group, if a father had completed two assessments, only the earlier assessment was retained. Among these fathers, 1,056 (65%) completed one assessment when their child was 24 months or younger, 415 (26%) completed two assessments, and 144 (9%) completed three assessments within this age range. After removing the second assessments, in total, 2,318 depression screening assessments were included (0–6 months: *n* = 597; 7–12 months: *n* = 701; 13–18 months: *n* = 485; 19–24 months: *n* = 535), representing 1,615 fathers. At T1, fathers were on average 33.41 years old (*SD* = 4.44), and 57.68% resided in urban areas. Regarding household gross monthly income, 12.91% reported a family income below 45,000 SEK, 63.94% reported an income between 45,000 and 84,999 SEK, and 23.15% reported an income of 85,000 SEK or higher.

### Measures

The EPDS was used to assess depression symptoms (Cox et al., 1987). The scale consisted of 10 items and has been validated for assessing paternal depression symptoms (Shafian et al., 2022). Fathers reported their mood and feelings over the past 7 days (e.g., “I have felt sad or miserable”) on a 4-point scale ranging from 0 to 3, yielding total scores ranging from 0 to 30. Meta-analyses have shown that EPDS total scores of 7 to 10 or higher detect paternal postnatal depression (Shafian et al., 2022). The internal consistency, as measured via McDonald’s Omega, of the total scale, based on the four infant age groups, ranged between .90 and .94 in this study.

### Data Analysis

This study aimed to examine the three-factor structure of the EPDS proposed by Coates et al. (2017) in mothers and Cockshaw et al. (2023) in fathers, in a sample of Swedish fathers of children aged 0 to 24 months. Means and standard deviations were calculated for each EPDS item. Internal consistency was assessed using McDonald’s Omega values for each of the three subscales in the proposed three-factor model, as well as for the whole scale.

The three-factor structure of EPDS was tested using confirmatory factor analysis (CFA). As EPDS uses a 4-point response scale, and the data were not normally distributed (*p*s < .05 for all items according to Shapiro–Wilk tests), indicators were treated as ordinal. We used weighted least square mean and variance adjusted (WLSMV) estimator and “theta” parameterization in the “lavaan” package. We also examined the one-factor and two-factor structures that were previously evaluated by Coates et al. (2017) and Cockshaw et al. (2023), and compared their model fit indices with those of the three-factor model. Model fit indices examined include comparative fit index (CFI), Tucker–Lewis index (TLI), root mean square error of approximation (RMSEA), and standardized root mean square residual (SRMR). With ordinal data, we reported scaled CFI, TLI, and RMSEA, as well as standard SRMR. The following criteria were used to evaluate model fit: CFI and TLI values greater than .95 suggest good fit (Hu & Bentler, 1999); RMSEA values smaller than .060 and .080 suggest good and acceptable fit, respectively (Browne et al., 1993; Hu & Bentler, 1999); and SRMR values lower than .080 indicate a good fit (Hu & Bentler, 1999).

Measurement invariance of the EPDS across the four child age bands was tested. Although the EPDS was answered on a 4-point scale ranging from 0 to 3, the highest rating of some indicators (e.g., Item 9, crying) was rarely or never endorsed by fathers in certain age groups (see Supplemental Table A). Consequently, we merged ratings of 2 and 3 into a single level, making all indicators ternary. With ordered ternary data, the configural model is equivalent to the threshold invariance model (Wu & Estabrook, 2016). Based on this baseline model, we tested weak invariance by imposing equality constraints on loadings across groups (i.e., thresholds and loadings constrained). Strong invariance was tested by additionally constraining the intercepts (i.e., thresholds, loadings, and intercepts constrained), and strict invariance was tested by further constraining the residual variance (i.e., thresholds, loadings, and intercepts, residuals constrained). Because the WLSMV estimator was used, we conducted robust χ^2^ difference test using “satorra.2000” method in lavaan. We also calculated the changes in model fit indices.

As a supplementary analysis, we conducted the same CFA and measurement invariance tests, treating the indicators as continuous and without merging responses. In addition, because primiparous and multiparous fathers might experience postnatal depression differently, we examined measurement invariance across primiparous and multiparous parents as another supplementary analysis.

We calculated the percentage of fathers who screened positive for postnatal depression (EPDS total score ≥ 10) across the four age bands, using the original (unmerged) responses. Means and standard deviations of the three EPDS subscales were computed for non-depressed fathers (EPDS total score < 10), depressed fathers (EPDS total score ≥ 10), and all fathers, separately by age band. We compared fathers from different age bands on their EPDS subscale scores and total scores using analysis of variance (ANOVA). When a significant difference was observed, post hoc comparisons were performed using either Tukey’s Honestly Significant Difference (HSD) test or the Games–Howell test, depending on whether variances were heterogeneous.

Data were analyzed using R4.4.3/R Studio. Confirmatory factor analysis and measurement invariance test were conducted using the “lavaan” package (Rosseel, 2012).

## Results

The means and standard deviations for each item, as well as McDonald’s Omega coefficients for the three subscales and the total EPDS score, are presented in Table 1. All three subscales and the whole scale demonstrated good internal consistency.

**Table 1.** EPDS Item Descriptive Statistics and Scale Reliability.

Months	0–6 months (*n* = 597)		7–12 months (*n* = 701)		13–18 months (*n* = 485)		19–24 months (*n* = 535)	
Items	*M*	*SD*	*M*	*SD*	*M*	*SD*	*M*	*SD*
Item 1	0.44	0.62	0.45	0.62	0.50	0.66	0.49	0.67
Item 2	0.29	0.59	0.32	0.59	0.35	0.63	0.37	0.64
Item 3	0.81	0.87	0.85	0.91	0.96	0.93	0.80	0.89
Item 4	1.21	0.90	1.27	0.89	1.36	0.91	1.29	0.89
Item 5	0.33	0.65	0.40	0.72	0.46	0.78	0.37	0.69
Item 6	1.17	0.77	1.19	0.74	1.22	0.80	1.20	0.82
Item 7	0.45	0.72	0.45	0.74	0.57	0.79	0.49	0.73
Item 8	0.68	0.72	0.77	0.74	0.90	0.78	0.85	0.80
Item 9	0.19	0.44	0.24	0.48	0.32	0.53	0.27	0.53
Item 10	0.09	0.37	0.15	0.48	0.15	0.48	0.17	0.53
ANH	0.73	1.07	0.76	1.08	0.85	1.18	0.86	1.2
ANX	3.52	2.39	3.71	2.46	3.99	2.66	3.64	2.43
DEP	1.40	1.73	1.61	1.95	1.94	2.05	1.78	2.07
EPDS	5.65	4.44	6.08	4.77	6.78	5.13	6.27	4.87
ω_ANH_	.75		.76		.83		.83	
ω_ANX_	.79		.81		.83		.79	
ω_DEP_	.80		.83		.84		.83	
ω_EPDS_	.90		.91		.94		.92	

*Note*. ANH = anhedonia; ANX = anxiety; DEP = depressive affect; EPDS = Edinburgh Postnatal Depression Scale.

The model fit indices for the CFA models are presented in Table 2. The three-factor model demonstrated good fit across all four age bands. All scaled CFI and TLI values exceeded .95, and all SRMR values for the three-factor model were below .060, indicating good model fit. Although all scaled RMSEA values were above .060, they were below .080 for the three-factor model in three of four age bands. Across all age groups, the three-factor model showed better fit than the one-factor and two-factor models.

**Table 2.** Model Fit Assessing Four EPDS Structures in Fathers With Children at Four Age Bands.

Factor structure	Age group	Scaled χ^2^	df	*p*	CFI	TLI	RMSEA	RMSEA 90% CI	SRMR
Three factors: anhedonia (1−2), anxiety (3−6), and depressive affect (7−10)	0–6 months	132.31	32	<.001	.981	.973	.073	[.060, .086]	.053
	7–12 months	205.35	32	<.001	.972	.961	.088	[.077, .100]	.052
	13–18 months	126.97	32	<.001	.982	.975	.078	[.064, .093]	.052
	19–24 months	120.94	32	<.001	.983	.976	.072	[.059, .086]	.050
Two factors: anhedonia (1−2) and general distress (3−10)	0–6 months	191.06	34	<.001	.970	.960	.088	[.076, .100]	.061
	7–12 months	313.52	34	<.001	.956	.941	.108	[.098, .120]	.062
	13–18 months	175.12	34	<.001	.973	.964	.093	[.079, .106]	.058
	19-24 months	197.70	34	<.001	.968	.958	.095	[.082, .108]	.063
Two factors: anxiety (3−5) and depression (1−2, 6−10)	0–6 months	229.96	34	<.001	.963	.951	.098	[.087, .111]	.070
	7–12 months	181.41	34	<.001	.977	.969	.079	[.068, .090]	.050
	13–18 months	254.97	34	<.001	.958	.944	.116	[.103, .129]	.078
	19–24 months	242.88	34	<.001	.959	.946	.107	[.095, .12]	.072
One factor: all 10 items	0–6 months	335.38	35	<.001	.943	.927	.120	[.108, .132]	.083
	7–12 months	385.77	35	<.001	.944	.928	.120	[.109, .131]	.070
	13–18 months	382.89	35	<.001	.934	.915	.143	[.131, .156]	.093
	19-24 months	388.45	35	<.001	.931	.911	.138	[.125, .150]	.092

**Figure 1.**
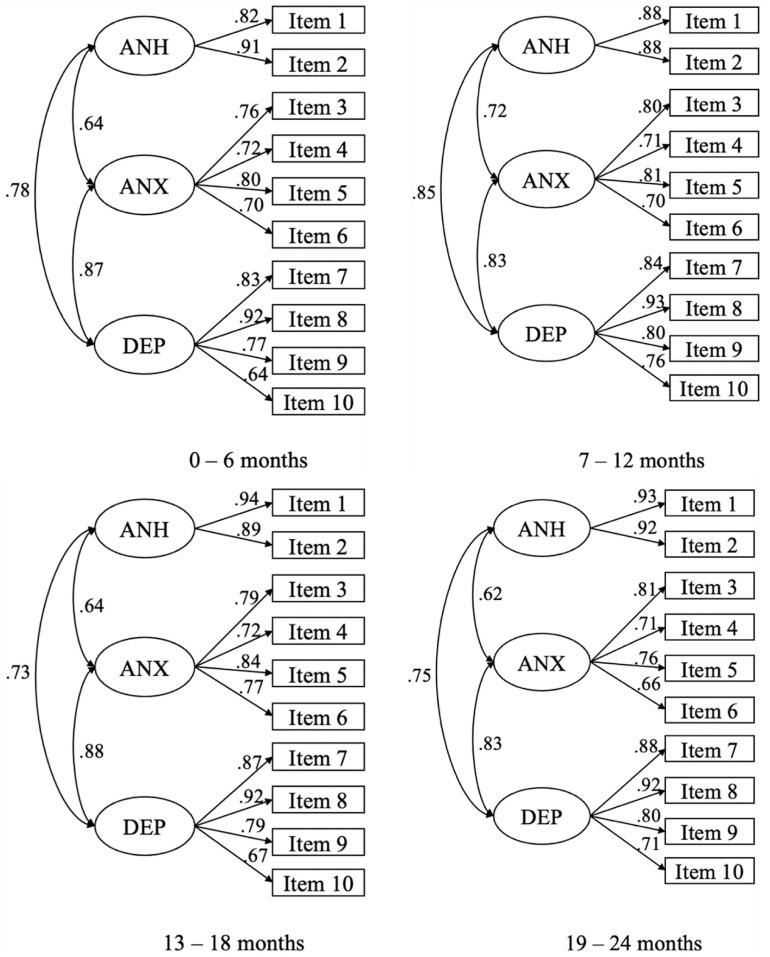
Three-factor models for fathers of children in four age bands. *Note*. ANH = anhedonia; ANX = anxiety; DEP = depressive affect.

The standardized factor loadings and factor covariances for the three-factor model are presented in Figure 1. Across the four age bands, all EPDS items loaded significantly onto their respective factors (all *p*s < .001). All items had standardized loadings above .70, with a few exceptions where loadings ranged between .64 and .67: Item 10 among fathers of infants aged 0 to 6 months and 13 to 18 months, and Item 6 among fathers of infants aged 19 to 24 months. The three latent factors (i.e., anhedonia, anxiety, and depressive affect) were significantly and positively correlated with one another across all age groups (all *p*s < .001). The covariance between the anhedonia and anxiety factor (ranging from .62 to .72) appeared to be slightly lower than the covariance between the anhedonia and depressive affect factors (ranging from .73 to .85) and between the depressive affect and anxiety factors (ranging from .83 to .88).

The results of the measurement invariance analyses across age bands are reported in Table 3. The model fit indices and changes in fit indices supported strict invariance across the four age bands. The robust χ^2^ difference test indicated no significant differences between the baseline and weak invariance models (Δχ^2^ = 27.00, Δ df = 21, *p* = .171), between weak invariance model and strong invariance model (Δχ^2^ = 30.25, Δ df = 21, *p* = .087), and between strong invariance model and strict invariance model (Δχ^2^ = 33.37, Δ df = 30, *p* = .307). Scaled CFI and TLI increased, and scaled RMSEA decreased with more stringent models, supporting strict invariance. Standard SRMR increased with more stringent models, but the degrees of change were small (≤ .003). Overall, the results supported strict measurement invariance across age bands.

**Table 3.** Measurement Invariance Test Across Age Bands.

Model	χ^2^	*Scaled* χ^2^	*df*	CFI	TLI	RMSEA	SRMR	*p* value of robust Δχ^2^ test	ΔCFI	ΔTLI	ΔRMSEA	ΔSRMR
Configural	309.28	500.52	128	.981	.974	.071	.055	-	-	-	-	-
Weak	326.78	520.90	149	.981	.977	.066	.055	.171	.000	.003	-.005	.000
Strong	355.98	514.01	170	.983	.982	.059	.056	.087	.002	.005	-.007	.001
Strict	393.14	491.82	200	.985	.987	.050	.059	.307	.002	.005	-.009	.003

*Note.* In this, and only in this set of analysis, responses of 2 and 3 of all items in Edinburgh Postnatal Depression Scale were merged as 2, resulting in three-level response scales.

Treating EPDS items as continuous similarly supported the three-factor structure (see Supplemental Table B) and at least strong measurement invariance (see Supplemental Table C). The EPDS demonstrated strict invariance across primiparous and non-primiparous fathers, and the results are reported in Supplemental Table D.

Using a cut-off score of 10 on the EPDS total score, 16.75% of fathers in the 0 to 6 months group, 20.26% in the 7 to 12 months group, 26.39% in the 13 to 18 months group, and 22.99% in the 19 to 24 months group met the criterion for at least mild depression. The means and standard deviations of three subscales of EPDS for non-depressed fathers, depressed fathers, and all fathers across the four age bands are shown in Table 4. Fathers of 13- to 18-month-old children reported higher anxiety and depressive affect than those of 0- to 6-month-olds. Depressive affect was also higher in fathers of 19- to 24-month-old children (vs. 0- to 6-month-olds) and in fathers of 13- to 18-month-old children (vs. 7- to 12-month-olds).

**Table 4.** The Means and Standard Deviations of Subscale Sum Scores in Fathers With EPDS Total Scores <10 versus ≥10 and in All Fathers.

Age bands		*M* (*SD*)											
		EPDS total score < 10				EPDS total score ≥ 10				All fathers			
		ANH	ANX	DEP	EDPS	ANH	ANX	DEP	EDPS	ANH	ANX	DEP	EPDS
0–6 months^a^		0.45 (0.78)	2.80 (1.78)	0.80 (0.99)	4.06 (2.61)	2.07 (1.31)	7.10 (1.74)	4.38 (1.55)	13.55 (2.98)	0.73 (1.07)	3.52 (2.39)	1.40 (1.73)	5.65 (4.44)
7–12 months^b^		0.41 (0.68)	2.86 (1.78)	0.87 (1.01)	4.14 (2.59)	2.16 (1.23)	7.06 (1.80)	4.50 (2.06)	13.72 (3.53)	0.76 (1.08)	3.71 (2.46)	1.61 (1.95)	6.08 (4.77)
13–18 months^c^		0.45 (0.74)	2.83 (1.74)	1.00 (1.11)	4.28 (2.69)	1.98 (1.42)	7.22 (2.05)	4.55 (1.81)	13.76 (3.67)	0.85 (1.18)	3.99 (2.66)	1.94 (2.05)	6.78 (5.13)
19–24 months^d^		0.44 (0.73)	2.71 (1.66)	0.92 (1.09)	4.08 (2.56)	2.24 (1.43)	6.74 (2.01)	4.63 (2.01)	13.61 (3.31)	0.86 (1.20)	3.64 (2.43)	1.78 (2.07)	6.27 (4.87)
ANOVA	*F*	0.468	0.665	2.878	0.574	0.869	1.208	0.331	0.075	1.848	3.449	7.774	5.146
	*df*	3, 1820	3, 1819	3, 1819	3, 1820	3, 490	3, 489	3, 489	3, 490	3, 2314	3, 2312	3, 2312	3, 2314
	*p*	.705	.574	.035	.632	.457	.306	.803	.973	.136	.016	<.001	0.002
Post hoc		-	-	c > a	-	-	-	-	-	-	c > a	c > a, d > a, c > b	c > a

*Note.* The anhedonia subscale sum scores range from 0 to 6; the anxiety and depressive affect subscale sum scores range from 0 to 12. ANH = anhedonia; ANX = anxiety; DEP = depressive affect; EDPS = total sum score of Edinburgh Postnatal Depression Scale.

a Indicates the means for fathers in the 0–6 months age band.

b Indicates the means for fathers in the 7–12 months age band.

c Indicates the means for fathers in the 13–18 months age band.

d Indicates the means for fathers in the 19–24 months age band.

For example, in the fourth column, c > a suggests that the mean for depressive affect was higher in the 13–18 months age band than in the 0–6 months age band.

## Discussion

Since questions persist about the dimensional structure of EPDS in fathers and whether scores are comparable as children age through infancy and toddlerhood, our study assesses these gaps by testing the EPDS factor structure and measurement invariance across four child-age bands in a national cohort of Swedish fathers. We found that a three-factor model of the EPDS: anhedonia (Items 1–2), anxiety (Items 3–6), and depressive affect (Items 7–10) consistently outperformed one- and two-factor alternatives across four child age bands (0–6, 7–12, 13–18, and 19–24 months). Model fit indices were acceptable to good at every age band, and strict invariance was supported across bands. Internal consistency was acceptable to good for all three subscales and excellent for the total scale. Prevalence based on a total score cut-off of ≥10 varied by age and peaked at 13 to 18 months (26.39%) in this cohort.

### Factor Structure of EPDS

Our results converge with previous research showing that a three-factor EPDS structure is often the most fitting, with anhedonia, anxiety, and depressive affect clusters reproducible across perinatal timepoints (Coates et al., 2017; Cockshaw et al., 2023; Reichenheim et al., 2011). At the same time, item behavior can vary across contexts such as postpartum timing, population/cultural setting, and analytic specification. For example, in an Italian cohort assessed immediately postpartum versus 3 months later, symptom change was driven primarily by the anxiety items (3–6), which decreased markedly over time, whereas the anhedonia items (1–2) showed little change; the same study also noted that Item 6 tends to load less consistently on the anxiety factor across studies (Petrozzi & Gagliardi, 2013). In a Japanese postpartum sample, the composition of the “depression” factor differed depending on the acceptable model, and prior cross-cultural work was noted to show inconsistent loading for Item 6 across countries; reverse-scored items (1–2) were also discussed as a potential source of clustering (Kubota et al., 2014). A systematic review of EPDS validation studies documents substantial heterogeneity in diagnostic performance across languages, timing of administration, and study methods, underscoring the risk of assuming comparability across settings (Gibson et al., 2009); accordingly, we test measurement invariance rather than assume it a priori.

Item-level patterns in our sample resonate with prior findings in fathers. Consistent with earlier studies (Cockshaw et al., 2023; Loscalzo et al., 2015; Massoudi et al., 2013), fathers in our cohort infrequently endorsed Item 9 (crying) and Item 10 (self-harm) across age bands. Cockshaw et al. (2023) reported that gender-based non-invariance between mothers and fathers was driven primarily by Item 9, suggesting that crying may function differently across genders. In the present study, Item 9 demonstrated good factor loadings despite its low mean endorsement. Thus, lower endorsement of Item 9 reflects response frequency rather than irrelevance of the indicator in fathers and aligns with Cockshaw et al. (2023) suggestion that male EPDS cut-points may need to be lower than female cut-points.

Given this, a central interpretive question is whether EPDS subscales should be used in addition to, or instead of, the total score when screening or modeling paternal mental health. In our data, a three-factor solution fit well, similar to Cockshaw et al. (2023), and achieved strict invariance across child-age bands, and the latent anhedonia, anxiety, and depressive affect factors were positively and substantially inter-correlated, consistent with facets of a broader general distress construct. This pattern supports continued use of the total EPDS score as an efficient screen in fathers, while permitting subscale-level exploratory analyses (e.g., whether anhedonia or anxiety relates differentially to family processes), provided subscale correlations are acknowledged and small, shared differences are not over-interpreted.

### Age-Patterning of Paternal Symptoms and Implications

The age-varying prevalence we observed, which was highest in the 13- to 18-month age band, extends prior meta-analytic work showing that paternal depressive symptoms often rise after the early postpartum period, with many studies identifying 3 to 6 months as a peak window (Paulson & Bazemore, 2010; Rao et al., 2020). A recent Swedish register-based cohort observed that fathers’ incidence of clinically diagnosed depression increased toward the end of the first postpartum year (Xiang et al., 2026). An important contextual detail is that these studies did not typically assess paternal depression beyond 12 months, meaning later-emerging peaks could not be detected in the aggregated evidence. In parallel, maternal evidence indicates that depression in the second postpartum year (13–24 months) remains common, with pooled prevalence similar to the first year, suggesting that risk persists beyond 12 months (Hellyer et al., 2025). Our finding of a pronounced elevation during toddlerhood is therefore filling a gap in paternal mental health research.

In the Swedish context specifically, a later-peaking symptom profile is also plausible on substantive grounds. Fathers commonly experience major role reorganizations during the second year of parenthood, including transitions into (1) being the primary parent when taking parental leave, (2) both parents returning to work after extended leave, and (3) children beginning preschool, where each of these transitions has been linked to elevated emotional strain or mental-health vulnerability in fathers. For example, evidence from multiple reviews shows that paternal leave can influence fathers’ mental health, with findings indicating that longer or structured leave can improve well-being, whereas fragmented or poorly supported leave can increase stress due to competing caregiving and employment demands (Philpott et al., 2022). Consistent with this, in a national Swedish longitudinal cohort, fathers who took 14 to 40 weeks of parental leave had substantially lower odds of screening positive for depression at follow-up than those taking 0 to 4 weeks, with dose–response models indicating that risk declined up to ~35 weeks of leave before plateauing (Wang & Wells, in press). When parents return to work, they may experience intensified work–family conflict, which has been identified as a contributor to psychological distress during the postnatal period (Cooklin et al., 2015). Research on the transition to parenthood indicates that balancing employment with the demands of infant and toddler care represents a period of heightened vulnerability for fathers’ mental health (Baldwin et al., 2018). In parallel, the period in which children begin preschool introduces changes in routines and caregiving expectations, contributing to family-level stress as parents reorganize responsibilities (DeCaro & Worthman, 2011).

Layered onto these contextual pressures is the fact that most screening for paternal depression occurs early in infancy, as reflected in the dominant evidence base: meta-analyses identifying the 3- to 6-month window as the peak period of paternal depressive symptoms were largely limited to studies sampling only up to 12 months postpartum (Paulson & Bazemore, 2010; Rao et al., 2020). This early infancy emphasis in the literature suggests that later-emerging symptom elevations, such as those we observe during toddlerhood, would have been overlooked. Taken together, these dynamics underscore a clear practical implication: screening only in the early postpartum period likely misses a sizable group of affected fathers, and father-inclusive surveillance beyond the first year is warranted in systems where caregiving responsibilities intensify during toddlerhood.

### Strengths and Limitations

The design of the present study leverages a large sample of Swedish fathers who completed the EPDS across the first two postnatal years, enabling direct comparisons of competing one-, two-, and three-factor structures across four child-age bands and formal measurement invariance testing across bands. Consistent with methodological recommendations for Likert-type ordinal indicators with few response categories, we treated EPDS items as ordered-categorical and re-estimated CFA and invariance models using WLSMV (Beauducel & Herzberg, 2006; Li, 2016). This modeling choice is important because maximum likelihood (ML) and full information maximum likelihood (FIML) approaches assume continuous indicators, whereas WLSMV is designed for ordinal indicators and often yields more accurate loading estimates under typical ordinal-data conditions (Beauducel & Herzberg, 2006; Li, 2016). At the same time, influential EPDS factor-structure studies in fathers have commonly analyzed EPDS items as continuous using maximum-likelihood approaches, which can contribute to differences in fit and parameter behavior across papers (Cockshaw et al., 2023; Massoudi et al., 2013). Importantly, after re-estimating our models with WLSMV, the three-factor structure remained the best-fitting solution across all age bands, with strong item loadings and excellent CFI/TLI and good SRMR across bands. Because absolute fit indices can vary by estimation method, we avoid interpreting “better fit” across estimators at face value and instead emphasize the stability of conclusions across specifications (Shi & Maydeu-Olivares, 2020). To further address potential estimator sensitivity, we conducted continuous-indicator sensitivity analyses without collapsing response categories, and the three-factor model again outperformed one- and two-factor alternatives across age bands, supporting robustness to common analytic choices.

A key interpretive consideration concerns sampling and generalizability: fathers were recruited via targeted Facebook advertisements (stratified by Swedish region) and the recruitment strategy intentionally sought to oversample fathers experiencing difficulties to ensure adequate analytic power, which reduces representativeness and may inflate screening-positive proportions relative to population-based sampling. Related Swedish work using similar Facebook recruitment and targeted advertisements has likewise noted that such recruitment can yield elevated depressive symptom frequencies, reinforcing caution in interpreting prevalence patterns as population rates (Wells & Aronson, 2021). However, the primary purpose of the present study is psychometric; evaluating factor structure and measurement invariance rather than estimating nationally representative prevalence, and the large sample strengthens precision for model comparison and invariance testing within the recruited population. Another interpretive limitation is that EPDS responses are self-reported and not validated against diagnostic interviews in this dataset; therefore, cut-offs should be interpreted as screening thresholds rather than diagnoses, consistent with prior father validation work and cautions about the EPDS capturing broad distress rather than depression alone in men (Massoudi et al., 2013).

Sparse endorsement of extreme response categories in certain age bands required collapsing upper response options for ordered-categorical invariance testing, a pragmatic step that facilitates estimation but may mask subtle differences at the highest symptom levels. We mitigated this concern by presenting continuous-indicator sensitivity analyses (without collapsing categories), which yielded substantively consistent conclusions regarding the preferred three-factor structure and invariance pattern. More broadly, because fit indices can shift as a function of estimator choice, we interpret absolute fit with estimator-appropriate expectations and prioritize the convergence of results across ordinal and continuous specifications (Shi & Maydeu-Olivares, 2020).

### Conclusions

In a Swedish cohort of fathers with children aged 0 to 24 months, the EPDS showed a replicable three-factor structure: anhedonia (items 1–2), anxiety (items 3–6), and depressive affect (items 7–10) that consistently outperformed one- and two-factor alternatives across all four child-age bands, with good-to-acceptable fit and strong internal consistency for the total score across bands. The factor structure was stable over time, with at least strong (and in the primary analyses, strict) measurement invariance across age bands, meaning that differences in total and subscale scores across 0 to 24 months can be interpreted as meaningful changes rather than artifacts of shifting measurement properties; importantly, the same three-factor structure also held regardless of parity, supporting comparable interpretation in primiparous and multiparous fathers. In practical terms, screening-positive proportions using EPDS ≥ 10 were non-trivial across the full 2-year window (16.75% at 0–6 months, 20.26% at 7–12 months, 26.39% at 13–18 months, and 22.99% at 19–24 months), underscoring the clinical rationale for father-inclusive screening that extends beyond the first postnatal year. Given the strong inter-correlations among the three latent factors, we recommend using the EPDS total score as the primary screening indicator in fathers, while reporting subscales as secondary descriptive indicators that may help characterize symptom profiles, and reserving any subscale-only case definitions for settings where they are validated against external criteria. Future research should test whether EPDS domains add incremental predictive value beyond the total score in father-focused screening pathways by linking factor/subscale patterns to external validators, including bonding, coparenting, work–family conflict, and child socio-emotional outcomes.

## Supplemental Material

sj-docx-1-jmh-10.1177_15579883261454722 – Supplemental material for Edinburgh Postnatal Depression Scale Factor Structure and Invariance in Fathers Across the First Two Postnatal Years: Evidence for a Three-Factor Model and Elevated Screening Positivity in Year 2 Supplemental material, sj-docx-1-jmh-10.1177_15579883261454722 for Edinburgh Postnatal Depression Scale Factor Structure and Invariance in Fathers Across the First Two Postnatal Years: Evidence for a Three-Factor Model and Elevated Screening Positivity in Year 2 by Jingyi Wang and Michael B. Wells in American Journal of Men's Health
