# Supplementary material for: Edinburgh Postnatal Depression Scale Factor Structure and Invariance in Fathers Across the First Two Postnatal Years: Evidence for a Three-Factor Model and Elevated Screening Positivity in Year 2
Source: Am J Mens Health. 2026 Jun 10;20(3):15579883261454722. doi: 10.1177/15579883261454722 (PMC13254417; doi:10.1177/15579883261454722)
Supplement: sj-docx-1-jmh-10.1177_15579883261454722 – Supplemental material for Edinburgh Postnatal Depression Scale Factor Structure and Invariance in Fathers Across the First Two Postnatal Years: Evidence for a Three-Factor Model and Elevated Screening Positivity in Year 2 [file sj-docx-1-jmh-10.1177_15579883261454722.docx]

**Supplemental Table A**

*Frequency of all indicators across age bands*

| **Age bands** | **All** | | | | **0-6 months** | | | | **7-12 months** | | | | **13-18 months** | | | | **19-24 months** | | | |
| --- | --- | --- | --- | --- | --- | --- | --- | --- | --- | --- | --- | --- | --- | --- | --- | --- | --- | --- | --- | --- |
|  | **0** | **1** | **2** | **3** | **0** | **1** | **2** | **3** | **0** | **1** | **2** | **3** | **0** | **1** | **2** | **3** | **0** | **1** | **2** | **3** |
| EPDS_1 | 1411 | 736 | 163 | 7 | 375 | 183 | 37 | 1 | 433 | 222 | 45 | 1 | 283 | 162 | 38 | 2 | 320 | 169 | 43 | 3 |
| EPDS_2 | 1711 | 467 | 115 | 20 | 460 | 105 | 25 | 6 | 518 | 145 | 30 | 5 | 353 | 98 | 31 | 3 | 380 | 119 | 29 | 6 |
| EPDS_3 | 1055 | 625 | 552 | 80 | 276 | 172 | 132 | 16 | 321 | 187 | 166 | 26 | 197 | 133 | 129 | 24 | 261 | 133 | 125 | 14 |
| EPDS_4 | 491 | 893 | 727 | 204 | 146 | 225 | 182 | 44 | 150 | 267 | 225 | 58 | 91 | 177 | 165 | 51 | 104 | 224 | 155 | 51 |
| EPDS_5 | 1697 | 372 | 212 | 33 | 452 | 97 | 42 | 6 | 510 | 107 | 74 | 8 | 338 | 82 | 52 | 12 | 397 | 86 | 44 | 7 |
| EPDS_6 | 455 | 1027 | 762 | 70 | 118 | 276 | 186 | 17 | 124 | 332 | 230 | 14 | 95 | 208 | 161 | 20 | 118 | 211 | 185 | 19 |
| EPDS_7 | 1521 | 504 | 258 | 32 | 405 | 125 | 60 | 7 | 479 | 139 | 71 | 11 | 292 | 117 | 66 | 9 | 345 | 123 | 61 | 5 |
| EPDS_8 | 912 | 1031 | 316 | 55 | 274 | 250 | 65 | 8 | 275 | 329 | 79 | 17 | 163 | 220 | 87 | 13 | 200 | 232 | 85 | 17 |
| EPDS_9 | 1796 | 458 | 60 | 2 | 495 | 91 | 11 | 0 | 546 | 137 | 17 | 0 | 342 | 129 | 12 | 1 | 413 | 101 | 20 | 1 |
| EPDS_10 | 2092 | 139 | 69 | 16 | 560 | 25 | 9 | 3 | 628 | 42 | 27 | 3 | 431 | 34 | 16 | 3 | 473 | 38 | 17 | 7 |

**Supplemental Table B**

*Model fit assessing four EPDS structures in fathers with children at four age bands (treating indicators as continuous).*

| **Factor structure** | **Age group** | **χ^2^** | **df** | ***p*** | **CFI** | **TLI** | **RMSEA** | **RMSEA 90% CI** | **SRMR** | **AIC** |
| --- | --- | --- | --- | --- | --- | --- | --- | --- | --- | --- |
| Three factors:  anhedonia (1−2),  anxiety (3−6), and  depressive affect (7−10) | 0-6 months | 107.42 | 32 | < .001 | .962 | .946 | .063 | [.050, .076] | .037 | 9825.28 |
|  | 7-12 months | 171.96 | 32 | < .001 | .948 | .927 | .079 | [.068, .091] | .041 | 11885.76 |
|  | 13-18 months | 105.37 | 32 | < .001 | .964 | .949 | .069 | [.054, .084] | .036 | 8591.29 |
|  | 19-24 months | 107.31 | 32 | < .001 | .963 | .948 | .066 | [.053, .080] | .041 | 9553.44 |
| Two factors:  anhedonia (1−2) and  general distress (3−10) | 0-6 months | 161.91 | 34 | < .001 | .935 | .914 | .079 | [.067, .092] | .043 | 9875.78 |
|  | 7-12 months | 258.39 | 34 | < .001 | .916 | .889 | .097 | [.086, .108] | .047 | 11968.20 |
|  | 13-18 months | 138.22 | 34 | < .001 | .948 | .932 | .080 | [.066, .094] | .039 | 8620.14 |
|  | 19-24 months | 177.61 | 34 | < .001 | .929 | .907 | .089 | [.076, .102] | .048 | 9619.74 |
| Two factors:  anxiety (3−5) and  depression (1−2, 6−10) | 0-6 months | 185.99 | 34 | < .001 | .922 | .897 | .087 | [.075, .099] | .048 | 9899.86 |
|  | 7-12 months | 193.52 | 34 | < .001 | .941 | .921 | .082 | [.071, .093] | .042 | 11903.33 |
|  | 13-18 months | 218.84 | 34 | < .001 | .908 | .879 | .106 | [.093, .119] | .050 | 8700.76 |
|  | 19-24 months | 231.88 | 34 | < .001 | .903 | .871 | .104 | [.092, .117] | .050 | 9674.00 |
| One factor: all 10 items | 0-6 months | 256.92 | 35 | < .001 | .887 | .854 | .103 | [.091, .115] | .054 | 9968.78 |
|  | 7-12 months | 324.55 | 35 | < .001 | .892 | .861 | .109 | [.098, .120] | .052 | 12032.35 |
|  | 13-18 months | 292.18 | 35 | < .001 | .873 | .836 | .123 | [.110, .136] | .058 | 8772.10 |
|  | 19-24 months | 339.68 | 35 | < .001 | .850 | .807 | .128 | [.115, .140] | .063 | 9779.81 |

**Supplemental Table C**

*Measurement invariance test across age bands (treating indicators as continuous).*

| **Model** | **χ^2^** | ***df*** | **CFI** | **TLI** | **RMSEA** | **SRMR** | **Δχ^2^** | **Δdf** | ***p*** | **ΔCFI** | **ΔTLI** | **ΔRMSEA** | **ΔSRMR** |
| --- | --- | --- | --- | --- | --- | --- | --- | --- | --- | --- | --- | --- | --- |
| Configural | 492.05 | 128 | .958 | .941 | .070 | .039 | / | / | / | / | / | / | / |
| Weak | 542.29 | 149 | .955 | .945 | .067 | .050 | 50.24 | 21 | <.001 | -.003 | .004 | -.003 | .011 |
| Strong | 568.46 | 170 | .954 | .952 | .064 | .051 | 26.17 | 21 | .200 | -.001 | .007 | -.003 | .001 |
| Strict | 662.02 | 200 | .947 | .952 | .063 | .062 | 93.56 | 30 | <.001 | -.007 | .000 | -.001 | .011 |

**Supplemental Table D**

*Measurement invariance test across parity*

| **Model** | **χ^2^** | ***Scaled* χ^2^** | ***df*** | **CFI** | **TLI** | **RMSEA** | **SRMR** | ***p* value of robust**  **Δχ^2^ test** | **ΔCFI** | **ΔTLI** | **ΔRMSEA** | **ΔSRMR** |
| --- | --- | --- | --- | --- | --- | --- | --- | --- | --- | --- | --- | --- |
| Configural | 217.68 | 350.51 | 64 | .980 | .972 | .075 | .054 | - | - | - | - | - |
| Weak | 220.90 | 353.98 | 71 | .980 | .975 | .071 | .055 | .622 | .000 | .003 | -.004 | .001 |
| Strong | 241.36 | 360.93 | 78 | .980 | .977 | .068 | .055 | .003 | .000 | .002 | -.003 | .000 |
| Strict | 249.42 | 325.26 | 88 | .983 | .983 | .058 | .056 | .686 | .003 | .006 | -.010 | .001 |

*Note.* In this set of analysis, responses of 2 and 3 of all EPDS items were merged as 2, resulting in 3-level response scales. Responses were treated as ordinal data.
